# How Might the Alcohol Minimum Unit Pricing (MUP) Impact upon Local Off-Sales Shops and the Communities Which They Serve?

**DOI:** 10.1093/alcalc/agt175

**Published:** 2013-11-28

**Authors:** Alasdair J.M. Forsyth, Anne Ellaway, Neil Davidson

**Affiliations:** 1 Institute for Society and Social Justice Research, Glasgow Caledonian University, G4 0BA Glasgow, UK; 2 MRC Social and Public Health Sciences Unit, University of Glasgow, G12 8RZ Glasgow, UK; 3 School of Law, University of Edinburgh, Old College, EH8 9YL Edinburgh, UK

## Abstract

**Aims:** The aim of the study was to assess the likely impact of the Scottish Government's proposed alcohol minimum unit pricing (MUP) policy on community off-sales outlets (convenience stores or corner shops), and, in turn, on the local people who purchase drinks at such premises. This research adds to our knowledge by linking sales of alcohol products which will be affected by MUP (e.g. at the proposed 50 ppu) to the types of communities where these are the ‘drinks-of-choice’. **Methods:** A survey of independent community off-sales operating within the city of Glasgow, Scotland (*n* = 271) returned 144 completed questionnaires enquiring about each shop's customer base, best-selling alcohol products and participating shopkeepers' views on MUP. Responses were measured against current alcohol product prices (i.e. whether potentially affected by MUP) and local levels of socio-economic deprivation. **Results:** Participating shopkeepers were divided in their support for MUP, although more were in favour than against. Support for MUP tended to be rooted in business concerns. A majority reported having at least one best-selling alcohol product which will be affected by the proposed MUP policy at current prices, with the beverages that would be most affected (e.g. white cider) tending to be best-sellers at shops serving deprived communities. **Conclusion:** MUP is likely to impact most in socio-economically deprived communities. This is also where alcohol-related health and other inequalities are currently greatest.

## INTRODUCTION

Alcohol minimum unit pricing (MUP) is a policy mandating the lowest retail price at which alcohol products can be sold (i.e. a floor-price). The level of MUP is determined by the cost per unit of alcohol (regardless of beverage type, i.e. unlike taxation). In the UK, a ‘standard unit’ of alcohol contains 10 ml, or 8 g, of pure ethanol. Thus, for example, if MUP was set at 30 pence per unit (30 ppu), then a drink containing 2 standard units of alcohol could not be sold to customers at a price below £0.60. The aim of MUP is to improve public health by lowering population-level consumption, and (in contrast to taxation) to target those groups most at-risk from alcohol-related health inequalities by affecting the cheaper beverages that they purchase (Stockwell *et al*., 2012; Chalmers *et al*., 2013).

On the 24 May 2012, the Scottish Government passed the *Alcohol (Minimum Pricing) (Scotland) Act 2012*, legislation to implement MUP across Scotland at 50 ppu (Scottish Government, 2012a,b). A legal challenge to this legislation by the Scotch Whisky Association was rejected by the Scottish courts on 3 May 2013. Although an appeal against the court's judgement has been made, it is possible that MUP could be implemented in the near future. Not all sections of the alcohol industry objected to this legislation. Some, most notably the Scottish Licensed Trade Association (SLTA) and several brewers were supportive (Scottish Government, 2011). Their support reflects price differentials between the on-trade (pubs, clubs and restaurants where alcohol is sold for immediate consumption on premises) and off-trade (shops where alcohol is sold for later consumption off premises) sectors. If implemented, MUP could make the on-trade sector more competitive (SLTA, 2011). Monitoring and Evaluating of Scotland's Alcohol Strategy (MESAS) estimates that the average price of alcohol on-trade is 153 ppu compared with 49 ppu off-trade (Robinson and Beeston, 2013).

MUP will affect problem drinkers because such consumers tend to choose alcohol brands offering the greatest value for money (Black *et al*., 2010). A customer survey by Crawford *et al*. (2012) found an interaction between alcohol consumption and household income, with persons of low income being more likely to purchase alcohol below 50 ppu if they scored higher on an alcohol use disorder identification test (AUDIT-C). Furthermore, it is believed that MUP will end what is known as ‘pocket money prices’ (Barton, 2013) which may divert younger consumers from becoming problem drinkers (Black *et al*., 2010).

In practice this means that MUP will impact upon less economically advantaged consumers (Meng *et al*., 2013). However, these are the very groups who are at risk of, or who already experience, the greatest levels of alcohol harms. For example, the rate of general acute inpatient discharges with an alcohol-related diagnosis in the most deprived geographical quintile of Scotland is more than seven times that of the least deprived quintile (Information Services Division, 2013). Nevertheless, a gap remains in our knowledge as to how the impact of MUP will be felt in the community. Alcohol consumption figures collated by Her Majesty's Revenue & Customs, or alcohol product retail sales figures collected by market analysts (e.g. that collected by *The Nielsen Company* for MESAS), provide no indication of where consumers live. Similarly, although drinks purchased at supermarkets can be recorded via customer survey or receipt data, these are unlikely to reflect local consumption patterns because such premises serve wider, diverse geographical areas, with purpose built car-parks indicating destination shoppers who do not live locally.

This research will help to fill the gap in our knowledge of MUP's likely affect on socially contrasting communities. This is achieved via data collected from an often overlooked group in the MUP debate, the small retailer or community off-sales shopkeeper. We gathered data from small off-trade outlets serving local customers, to assess how MUP will differentially affect such small businesses and their clientele depending on local variations in alcoholic beverage choice and area deprivation.

We chose to focus on these small retailers, and purposively screened-out supermarkets, department stores, service-stations, etc. There were four main reasons for this. Firstly, community convenience stores are known to be better indicators than supermarkets of local health-related context because of their greater ‘walkability’ which makes them more reflective of the local ‘nutritional environment’ (Minaker *et al*., 2013). We aimed to recruit shops serving customers within walking distance (i.e. a geographical community) rather than superstores, city centre or retail park outlets. Secondly, community shops offer counter-service, allowing the servers to know their local customers' beverage preferences. In contrast, supermarkets (their checkout operators, managers, etc.) are not generally representative of their locality (if indeed they have any local customers). Thirdly, we are interested in local beverage preferences (e.g. brands under 50 ppu) not global sales volumes/proportions. Only the ‘drinks-of-choice’ (best-sellers) purchased from community outlets can reflect local preferences, even if most local people buy most of their alcohol elsewhere. Fourthly, all independent small shops have an individual local expert (shopkeeper) who is able to participate in this research, something that might not be possible with chain stores or their employees.

It was hypothesized that cheaper alcohol products/brands (those to be affected by MUP) would be the most likely to be best-sellers at shops serving disadvantaged communities (i.e. which experience greater levels of alcohol-related harm).

## METHODS

The research involved a questionnaire survey of independent community off-sales operating in the city of Glasgow, Scotland (population circa 600,000) conducted between October 2011 and April 2012. Glasgow experiences some of the highest levels of socio-economic deprivation and alcohol-related problems in the UK (Hanlon *et al*., 2006; Leyland *et al*., 2007; NHS Greater Glasgow & Clyde, 2011). Further, alcohol use in this city is a major contributor to the ‘Glasgow effect’ (McCartney *et al*., 2011). That is, levels of health inequalities, mortality and morbidity in this city are beyond what might be anticipated from deprivation alone (Walsh *et al*., 2010). In contrast to Scotland's other major city, Edinburgh, Glasgow's alcohol problems have been described as less closely linked to social class, with problem drinkers being more likely to purchase their drinks from ‘independent outlets (corners shops)’, that is community off-sales as opposed to supermarkets (Chick and Gill, 2013).

Local levels of deprivation are measured here using the Scottish Index of Multiple Deprivation 2012 (SIMD2012) (Scottish Government, 2012c) for Census small areas known as Data Zones. The SIMD2012 is calculated from seven domains: ‘income’ (weighted at 27.9% of the total deprivation score), ‘employment’ (also weighted 27.9%), ‘health’ (weighted 14.0%), ‘education’ (14.0%), ‘geographic access’ (9.3%), ‘crime’ (4.7%) and ‘housing’ (2.3%). One of the seven indicators that make up the Health Domain of the SIMD2012 is ‘hospital stays related to alcohol use per person’ which is expressed as a standardized ratio (100 = mean for all Scotland). Data Zones are small geographical areas which lie within local government boundaries. There are 6505 Data Zones in Scotland (median population = 769). Where possible these have been shaped to respect physical boundaries and natural communities containing households with similar social characteristics. Each Data Zone is allocated a total SIMD2012 score, which is subdivided into quintiles, with Quintile-1 representing the most deprived areas. Glasgow represents the greatest concentration of these most deprived Data Zones in Scotland (26.2% of all Quintile-1). The Glasgow City local authority area is made up of 694 Data Zones, 341 (49.1%) of which score in the most deprived Quintile-1. The remainder comprise 131 (18.9%) Data Zones in Quintile-2, 87 (12.5%) in Quintile-3, 77 (11.1%) Quintile-4 and 58 (8.4%) in the least deprived Quintile-5.

Potential premises for participation in the research were first identified by using a database of 618 off-licensed addresses provided by the local authority. In order to check eligibility for inclusion as community off-sales, each of these addresses was visited by the research team. Any shops offering counter-service, operating as either licensed grocers or designated off-sales shops were deemed eligible for inclusion. By this method 271 premises were found to be currently operating as community off-sales. All were either small independent retailers (often named after the proprietor or the locality) or a licensed member of a ‘symbol’ group of convenience stores (e.g. *Spar*). This left 347 licensed addresses which were either non-applicable (*n* = 129) or non-eligible (*n* = 218) for inclusion in the survey. For example, the premise that was located in the most deprived Data Zone of all on the database of licensed addresses was excluded when it was identified as the gift shop of one of Glasgow's two major football team's stadium rather than a community shop.

All non-applicable addresses on this database comprised: 27 shops that were still trading but not selling alcohol, 87 shops that had ceased trading (62 of these were part of several designated off-licensed chain-stores that had recently gone into administration, e.g. *Haddows n* = 43), 13 demolished locations and 2 cases where adjacent shops had merged into a single premise. Non-eligible premises, which, although selling alcohol were excluded because they were not operating as community off-sales, comprised: 23 caterers, 23 wholesalers, 18 gift shops, 11 petrol/service-stations, 6 chemists, 5 department stores (e.g. *Debenhams*), 2 bookmakers and 84 supermarkets (including representatives of each the four UK ‘majors’, i.e. *Tesco n* = 11, *Sainsbury's n* = 8, *Morrison's n* = 7 and *ASDA n* = 5). In the field, licensed supermarkets were distinguished from community off-sales by whether they only offered counter-service only (included in the sample) or a row of checkouts (excluded from the sample). Finally, when recruiting for an earlier qualitative interview phase of the study, the head office of the one remaining designated off-sales chain operating in Glasgow opted not to permit any of their stores to participate in the research (*n* = 44) and so their stores were not included in the questionnaire survey phase. Although their refusal to participate was disappointing, their inclusion may have biased the sample by constituting a group of outliers (in the absence of any other chains, these having gone into receivership). This chain's premises were not uniformly distributed across Glasgow, in that they tended to be located in SIMD2012 Data Zones with significantly elevated levels of deprivation (e.g. the mean standardized ratio of ‘hospital stays related to alcohol’ for Zones with a chain-store was 268.7, compared with 207.0 for other addresses on the off-licensed database, *t* = 2.565, df = 53.210, *P* = 0.013).

Questionnaires were hand delivered to all of Glasgow's 271 eligible, independent community off-sales by the researchers, with an information sheet that explained the purpose and confidentiality of the study. A stamped addressed envelope was provided for the questionnaire's return. To maximize response rates the questionnaire was brief (two sides of A4). This asked participants about their shop's size (number of staff), their customer base (including a series of 5-point, multi-choice, Likert scales scored from ‘none’ to ‘all’), their perceptions of alcohol problems (compared with elsewhere in the city), how large a part of their business alcohol sales were, their views concerning MUP (whether in favour or against and why) and what their best-selling alcohol products were. This latter question was left open-ended (the example of ‘*McEwan's Lager*’ was given, which no participant cited as a best-seller), and from this information it was possible to ascertain which shops were most likely to be affected by MUP.

Price information about alcohol brands cited as best-selling products in the survey was obtained via the website http://www.mysupermarket.co.uk/ (accessed May 2013) which gives the average prices over the previous calendar year calculated across a number of retailers selling any particular major branded product including alcohol. Where an alcohol brand was not listed in *mySupermarket.co.uk* (either because it was an own-label item, Scotland specific brand or a niche product e.g. ‘tonic wine’) participating shops were revisited and comparable volume's prices were noted.

Alcohol brands were grouped into eight categories. In the first instance this was four basic product types: beer, cider, wine and spirits. These were then subdivided to allow mainly low cost, high strength sub-categories, each of which has been implicated in excessive alcohol-related harms. Specifically, super lager (or ‘super-strength beer’, Thames Reach, 2005), white cider (Black *et al*., 2010; Goodall, 2011), vodka (lower priced vodka has been implicated by MESAS as the driver of overall excess alcohol consumption in Scotland in comparison with England and Wales, Robinson and Beeston, 2013) and ‘other’ often fortified beverages such sherry or caffeinated/‘tonic’ wine (often linked to violent behaviour in Scotland, Galloway *et al*., 2007; Simpson and Pearson, 2012).

## RESULTS

More than half, 144 (53.1%), of the 271 shops eligible to participate in the research returned a questionnaire (45.7% without the exclusion of the non-participating chain). The SIMD2012 Data Zone distribution of the 144 participating shops reflected Glasgow's high levels of deprivation, comprising 67 (46.5%) shops located in Quintile-1 Data Zones, 35 (24.3%) in Quintile-2, 24 (16.7%) in Quintile-3, 12 (8.3%) in Quintile-4 and 6 (4.2%) in Quintile-5. The corresponding figures for the 144 non-participating community off-sales (who received questionnaire but did not complete it) were Quintile-1 66 (52.0%), Quintile-2 25 (19.7%), Quintile-3 21 (16.5%), Quintile-4 8 (6.3%) and Quintile-5 7 (5.5%), a non-significant difference (chi-square = 1.691, df = 4, *P* = 0.792). Thus the final sample was broadly representative of independent community off-sales provision by area deprivation in the city.

Questionnaire responses confirmed that participating shops were, as intended, small businesses (as opposed to superstores), with a mean self-reported staffing level of 3.1 persons (*n* = 143, range 1–14). Table 1 shows participants' responses to four Likert scales asking about their shops' customer base.

**Table 1.** Shopkeeper's customer base as a proportion of local and non-local people

Customer type	‘All’	‘Most’	‘Many’	‘Few’	‘None’
Locals (walking distance) (*n* = 142)	14	94	28	4	2
Cumulative %	9.9	76.1	95.8	98.6	100
Destination shoppers (*n* = 141)	1	6	11	64	59
Cumulative %	0.7	5.0	12.8	58.2	100
Students or tourists (*n* = 140)	1	13	26	67	33
Cumulative %	0.7	10.0	28.6	76.4	100
Passing trade (*n* = 141)	1	13	48	72	7
Cumulative %	0.7	9.9	44.0	95.0	100

From Table 1 it can be seen that the proportion of customers who could be described as ‘locals (walking distance)’ was much greater than the proportions described as ‘passing trade’, ‘students or tourists’ (i.e. non-local residents) or ‘destination shoppers (come here from other areas)’. For example, only six participants (4.2%) stated that ‘few’ or ‘none’ of their customers lived within walking distance, options which constituted the majority of responses for every other given customer type. Unsurprisingly, these community shopkeepers reported a high degree of familiarity with their clientele. When asked ‘What percentage of customers do you know well (by name, age etc.)?’ a mean of 59.4% (*n* = 142, SD = 26.1) was recorded. Thus participating shopkeepers were serving customers who tended to live within their local community and who they often knew personally.

A three-point multi-choice question invited respondents to rate alcohol problems in their neighbourhood in comparison with ‘Glasgow as a whole’, with the options provided being ‘not as bad’ (selected by 46 respondents), ‘about the same’ (by 77 respondents) and ‘worse’ (*n* = 16 respondents). When these responses were compared with the SIMD2012 data measuring ‘hospital stays related to alcohol use per person’ for their shop's Data Zone, a statistically significant relationship was found (one-way ANOVA, F = 5.698, *P* = 0.004). Shopkeepers reporting that alcohol problems were ‘worse’ locally served in Data Zones with a mean standardized hospitalization ratio of 231.7, compared with 194.8 for those reporting local problems were ‘about the same’, and only 132.8 for ‘not as bad’. Thus even although ‘not as bad’ (Glasgow) Data Zones scored 32.8% above the Scottish national average, respondents had sufficient local knowledge to accurately differentiate the levels of alcohol problems within their communities.

When asked what ‘percentage of your total sales would you estimate are alcohol products’, the mean answer was 48.4% (*n* = 139, SD = 26.2). Although this is just under half of total sales, participating shopkeepers reported a high degree of dependence on alcohol for their business survival, with 88 (61.5%) stating that their shop could not survive without a license. Only 11 (7.7%) stated they could survive ‘easily’ without their license, with the remaining 44 (30.8%) indicating that their shop could survive but ‘with difficulty (e.g. cost jobs)’. When asked ‘Do you feel businesses like yours are under threat from the major supermarkets?’ 112 (78.3%) indicated ‘yes’, compared with only 31 (21.7%) who said ‘no’.

Responding shopkeepers were asked directly whether or not they were ‘supportive of the proposed alcohol minimum pricing policy?’ A majority, 85 (59.9%), indicated ‘yes’ they were supportive of MUP, compared with 52 (36.6%) who indicated ‘no’. Five (3.5%) respondents wrote ‘don't know’ on their questionnaire beside this question, despite no such option being provided. A supplementary open-ended question offered respondents the opportunity to provide their reasons for supporting MUP or not. From this it was possible to code responses, for supporting MUP or not, under two broad themes, as either ‘business reasons’ or as ‘social-responsibility reasons’ (i.e. any community, socio-economic, crime or public health issues). These are summarized by Table 2 (more than one reason could be given by each shopkeeper).

**Table 2.** Community shopkeepers' reasons for supporting or not supporting MUP

Support MUP		Not support MUP	
Any business reasons	45		21
Affect supermarkets	32	Not affect Supermarkets	6
Level the playing field	17	Affect small retailer	5
Help small retailer	7	Help supermarkets	5
Not affect sales	3	Not affect sales	4
Other business reason	1	Harm profits	3
		Other business reason	3
Any community reasons	22		25
Help alcohol problems	8	Not help alcohol problems	12
Less drunken crime	5	Not reduce overall consumption	4
Affect under-ager drinkers	5	More theft crime	4
Reduce overall consumption	3	Affect responsible drinkers	3
Other community reason	4	Affect working class drinker	3
		Harm national economy	2
		Other community reason	1

As can be seen from Table 2, shopkeepers who supported MUP tended to do so for ‘business reasons’ (45 respondents who were in favour of MUP gave at least one ‘business reason’ for their support, compared with only 22 who gave a ‘social-responsibility reason’). In contrast to what might be anticipated, ‘business reasons’ for supporting MUP did not relate directly to any revenue gain which MUP might generate for them personally. Instead, ‘business reasons’ (including those for being against MUP) tended to relate to how the policy might affect the supermarkets. The five respondents who stated that they did not know whether they supported MUP are not represented on Table 2, although 2 of these stated that their support depended upon how the policy would affect the supermarkets.

Alcohol products which the participating shopkeepers cited as their best-sellers are shown in Table 3 (more than one beverage could be cited by each shopkeeper). The first column of Table 3 shows every beverage or brand cited as a best-seller by at least one responding shopkeeper. The second column indicates the number of shops where this product was cited as a best-seller. Thirty-three specific drinks brands were cited. The brand cited most often was *Tennent's Lager* (a best-seller at 85 shops). This is a local beer, which at the time of the survey sponsored both the country's largest music festival and Glasgow's two major football teams. The remainder of Table 3 calculates/estimates the ppu for each alcohol brand/product. Where possible, the volume of each alcohol products' brands were standardized as follows: spirits to 100 cl glass bottles, wines to 75 cl glass bottles, white cider to 200 cl plastic containers, and beers or amber cider to four packs of 44 cl cans.

**Table 3.** Best-selling alcohol products at Glasgow's community off-sales

Best-selling product/brand	*n*	Price source	Calculation of cost per unit	Unit cost
Beer (ordinary strength ale and lager)				
*Tennent's Lager*	85	Symbol	£4.50/4 pack 50 cl (cans) at 4.0% = 8.0 units	56.3p
*Budweiser*	23	Website	£4.26/4 pack 44 cl (cans) at 4.8% = 8.5 units	50.1p
*Stella Artois*	11	Website	£4.17/4 pack 44 cl (cans) at 4.8% = 8.5 units	49.1p
*Carlsberg*	2	Website	£3.73/4 pack 44 cl (cans) at 3.8% = 6.7 units	55.7p
*Carling*	2	Website	£3.46/4 pack 44 cl (cans) at 4.0% = 7.0 units	49.4p
*Kronenbourg*	1	Website	£4.25/4 pack 44 cl (cans) at 5.0% = 8.8 units	48.3p
*Foster's*	1	Website	£3.66/4 pack 44 cl (cans) at 4.0% = 7.0 units	52.3p
*Skol Lager*	1	website	£2.59/4 pack 44 cl (cans) at 2.8% = 4.9 units	52.9p
Own-label	1	Label shop	£4.75/4 pack 44 cl (cans) at 5.0% = 8.8 units	54.0p
*Furstenberg*	1	Participant	£1.75/50cl (bottle) at 5.3% = 2.7 units	66.8p
‘Scottish Beers’	1	Local ‘craft’ bottled beers = assume over 50 p per unit		
‘Speciality Beers’	1	Imported Mexican bottled beers = assume over 50p per unit		
‘Beer’ or ‘lager’	10	Excluded as insufficient information establish if price affected by MUP		
Super lager (extra-strength beer)				
*Tennent's Super*	6	Website	£7.20/4 pack 44 cl (cans) at 9.0% = 15.8 units	45.5p
*Skol Super*	1	Participant	£7.99/4 pack 50 cl (cans) at 9.0% = 18.0 units	44.4p
‘Super lagers’	1	As for all other super lagers = assume between 40p and 50p per unit		
Cider (ordinary strength amber cider)				
*Strongbow*	17	Website	£3.65/4 pack 44 cl (cans) at 5.0% = 8.8 units	41.5p
*Magners*	2	Website	£4.15/4 pack 44 cl (cans) at 4.5% = 7.9 units	52.5p
*Scrumpy Jack*	1	Symbol	£1.50/50 cl (can) at 5.0% = 3.0 units	50.0p
*Blackthorn*	1	Independent	£1.09/50 cl (can) at 5.0% = 2.5 units	43.6p
‘Ciders’	5	Excluded as insufficient information establish if price affected by MUP		
White cider				
*Frosty Jack*	21	Symbol	£3.55/200 cl (plastic bottle) at 7.5% = 15 units	23.7p
*Pulse*	4	Independent	£2.99/200 cl (plastic bottle) at 7.5% = 15 units	19.9p
*White Star*	1	Participant	£3.55/200 cl (plastic bottle) at 7.5% = 15 units	23.7p
*Diamond White*	1	Participant	£3.59/200 cl (plastic bottle) at 7.5% = 15 units	23.9p
‘White ciders’	1	As for all other white ciders = assume under 30p per unit		
Spirits (other than vodka)				
*Whyte & Mackay*	2	Website	£18.86/100 cl (bottle) at 40.0% = 40.0 units	47.2p
*Grouse*	1	Website	£21.61/100 cl (bottle) at 40.0% = 40.0 units	54.0p
*Jack Daniel's*	1	Website	£22.61/100 cl (bottle) at 40.0% = 40.0 units	56.5p
own-label whisky	1	Label shop	£17.15/100 cl (bottle) at 40.0% = 40.0 units	42.9p
*Havana Rum*	1	Website	£17.29/70 cl (bottle) at 40.0% = 28.0 units	61.8p
‘Spirits £30 plus’	1	Self-reported price = assume over £0.75p per unit		
‘Single malts’	1	Self-reported will not be affected by MUP = assume over 50p per unit		
‘Tequila’	1	As with all brands on website = assume over 50p per unit		
Vodka				
*Glen's*	48	Website	£14.44/100 cl (bottle) at 37.5% = 37.5 units	38.5p
*Smirnoff*	10	Website	£18.85/100 cl (bottle) at 37.5% = 37.5 units	50.3p
Own-label	1	Label shop	£17.47/100 cl (bottle) at 37.5% = 37.5 units	46.6p
‘Vodka’	4	Excluded as insufficient information establish if price affected by MUP		
Wine (ordinary strength table wine)				
*Blossom Hill*	6	Website	£5.84/75 cl (bottle) at 11.5% = 8.6 units	63.7p
*Echo Falls*	5	Website	£5.00/75 cl (bottle) at 12.5% = 9.4 units	53.2p
Own-label	2	Label shop	£4.99/75 cl (bottle) at 12.5% = 9.4 units	53.1p
*Hardys*	1	Website	£5.87/75 cl (bottle) at 12.5% = 9.4 units	62.5p
*Vinea*	1	Participant	£6.99/75 cl (bottle) at 14.0% = 10.5 units	66.6p
*Prosecco Ca'Rosa*	1	Participant	£10.00/75 cl (bottle) at 11.0% = 8.5 units	121.5p
‘Wine £8 to 10’	1	Self-reported price, assume 75cl (bottle) at 14% = 76.1 to 100.0p per unit		
‘Wine £10 bottle’	1	Self-reported price, assume 75 cl (bottle) at 14% = 71.4p per unit		
‘Speciality wine’	1	Imported Latin American wines = assume over 50p per unit		
‘Prosecco’	2	As with all brands on website = assume over 50p per unit		
‘[varied] wine’	22	As for all other wines = assume over 50p per unit		
Fortified wine and other beverages				
*Buckfast Tonic*	34	Independent	£7.00/75 cl (bottle) at 15.0% = 11.25 units	62.2p
*MD 20/20*	5	Independent	£7.00/75 cl (bottle) at 13.0% = 9.8 units	71.4p
*Mansion House*	2	Website	£3.33/70 cl (bottle) at 13% = 9.1 units	36.6p
‘Liqueurs’	1	Handmade British liqueurs = assume over 50p per unit		
‘Alcopops’	1	Excluded as insufficient information establish price per unit band		

The ppu for 20 of the 33 specific brands cited could be calculated directly from information gained via the *mySuper-market.co.uk* website alone. Note that the wine prices refer to each label's cheapest variety on the *mySupermarket* website (e.g. *Blossom Hill White* and *Echo Falls Fruity White*) though in practice even these were always above 50 ppu. Three of the remaining 13 brands that could not be priced via the *mySupermarket* website (including *Tennent's Lager*) were priced via observation in a branch of the symbol group whose shops participated most often in the survey (*n* = 15), located centrally beside Glasgow Caledonian University. Similarly, four brands not sold by the symbol store were priced by observation at a nearby independent store. This left six brands (all *n* = 1) where the individual shops which had cited these one-off answers as a best-seller were revisited to observe prices.

It was also possible to estimate MUP-relevant price information for several products where no specific brand was mentioned. This included four instances where an ‘own-label’ product was cited. These were priced via observations in the symbol store concerned. In three instances respondents self-reported a best-selling product's price, rather than citing a brand (all considerably greater 50 ppu). Another respondent stated that their speciality shop's best-selling (only) product ‘single malt whisky’ would not be affected by MUP. Indeed no malt whiskies on the *mySupermarket.co.uk* website were priced at a level that would be affected by MUP at 50 ppu. This was also the case for prosecco (*n* = 2 unbranded citations) and tequila (*n* = 1). In four instances, speciality (‘craft’ or ‘imported’) products cited as best-sellers were assumed to be (considerably) above 50 ppu (all *n* = 1, e.g. ‘handmade British liqueurs’). Finally, the generic beverage category answers ‘wine’ (*n* = 22), ‘super lagers’ (*n* = 1) and ‘white ciders’ (*n* = 1) were classified as either unaffected by MUP, between 40 ppu and 50 ppu, and under 30 ppu, respectively, because all specific brands in these categories for which a price could be calculated were consistently within these MUP level price-bands. However, it was not possible to estimate any price-band for the remaining (generic) answers ‘beer’ (best-seller at *n* = 10 shops), ‘cider’ (*n* = 5) and ‘vodka’ (*n* = 4) because the prices for individual brands of these products were found to vary across the 50 ppu threshold. Other answers excluded from further pricing analyses comprised the responses ‘any deal/offer’ (*n* = 2), ‘mixed’ (*n* = 2) and ‘alcopops’ (*n* = 1).

Of the 125 shops that provided sufficient information to estimate price-bands for their best-selling products, 75 (60.0%) had at least one best-seller which will be affected by MUP at the proposed 50 ppu level. If the MUP level was set at 40 ppu, the number of shops that would be affected was 64 (i.e. still around half), with 25 (20.0%) shops having a best-selling beverage that would be affected even at a 30 ppu level. It is these latter beverages (all white ciders sold in plastic, see Table 3) which will be affected the most by MUP at the 50 ppu level, their prices rising by at least 20p (or in one case over 30p) per unit.

When each shop location's Data Zone SIMD2012 score was compared with their cheapest best-selling alcohol product's price-band (i.e. whether above 50 ppu, if affected at the proposed 50 ppu MUP level, at the 40 ppu level or below 30 ppu), a statistically significant relationship was found. As is shown by Table 4, there was a tendency for shops located in more deprived quintiles to be more likely to cite a best-selling alcohol product in a lower price-band (χ^2^ = 22.850, df = 12, *P* = 0.029; likelihood ratio = 30.805, df = 12, *P* = 0.002; linear-by-linear association = 9.142, df = 1, *P* = 0.002). Only shops located in the two most deprived quintiles (Qunitile-1 or Quintile-2) cited any below 30 ppu products as best-sellers. It was noteworthy that there was no statistical association between support for MUP and best-selling product price-band, with 20.3% of supporters and 22.2% of non-supporters of MUP having a best-seller below 30 ppu, while 41.9% of supporters and 42.2% of non-supporters' best-sellers would be unaffected by MUP at 50 ppu.

**Table 4.** Cheapest best-selling alcohol product price band and deprivation quintile

Cheapest price/unit	Number of shops in each deprivation quintile (SIMD2012)				
	Quintile-1	Quintile-2	Quintile-3	Quintile-4	Quintile-5
Under 30p	17	8	0	0	0
30p to 40p	19	7	7	6	0
40p to 50p	2	4	3	1	1
Over 50p	19	13	9	4	5

It was also possible to compare each shop's best-seller price-band information with their local Data Zone's score for ‘hospital stays related to alcohol use per person’. According to SIMD2012 health domain data, the mean local ‘standardized hospitalization ratio’ for the 50 shops which had no best-sellers that would be affected by MUP was 170.6, for the 11 shops whose cheapest products were priced below 50 ppu but above 40 ppu this was 111.7, for the 39 shops whose cheapest best-seller was below 40 ppu but above 30 ppu this was 171.1, while for the 25 shops with a best-seller under 30 ppu this was 241.8 (one-way ANOVA *F* = 3.366, *P* = 0.021). That is, shops reporting the cheapest beverages (white cider) as a best-seller were located in neighbourhoods with higher levels of alcohol-related hospitalization.

## DISCUSSION

This research has successfully linked alcohol products, and whether or not they will be affected by MUP, to the communities where they are purchased. Three-quarters (76.1%) of participating shopkeepers stated that ‘most’ or ‘all’ of their customers lived within walking distance. This means that these shops' best-selling alcohol products reflect the preferred drinks-of-choice of the communities within which they were located. This localized approach contrasts with research into alcohol sales at macro-level (e.g. national level) which cannot link specific products to the communities who consume them. Similarly, analyses of supermarket receipts/sales will be compromised because even if such outlets are located in areas of disadvantage, they can attract a customer base of affluent, car owning, destination shoppers. Also, unlike our participants, who might be regarded as local experts in alcohol purchasing patterns, supermarket employees are less likely to know their customers personally, even where they live, let alone their drinking preferences or problems.

The main limitation of this research is that these findings are based on self-reports from small licensed shopkeepers, a group who clearly have a vested interest in alcohol issues. Three-fifths of respondents were supporters of MUP. Although this group mainly supported MUP for business reasons, it was noteworthy that this was because they felt that the policy would impact upon their rivals, the supermarkets, rather than because of any direct financial gain it may bring from alcohol sales. Ideally, the questionnaire could have asked respondents to self-report their own alcohol product prices. However, even as it stood, the question regarding best-sellers was the most sensitive in the questionnaire, arousing suspicion (perhaps non-participation) among shopkeepers, several of whom provided anecdotes to the research team about supermarkets sending agents into their shop to obtain such commercially important information. Instead we used an online price comparison website to estimate alcohol prices. The advantage of this was that it provides average prices over time across the UK for many products (i.e. a more objective/independent method than price observations in individual shops where prices are likely to fluctuate and where product ranges vary or are limited). The disadvantage is that the website uses supermarket rather than community shop prices. Given our participants' views, it could be argued that supermarket prices would be cheaper. However the cheapest drinks of all (white ciders) are a feature of independent stores. Further, recently published MESAS data (Robinson and Beeston, 2013; pp. 12–15) show a similar price hierarchy between beverage types to Table 3, with for example ciders retailing disproportionately more often under 30 ppu. MESAS states that 60% of off-trade alcohol in Scotland retails under 50 ppu (the MUP threshold), the same percentage as shops reporting best-selling brands under 50 ppu in our calculations.

This research has found that the cheapest alcohol products were more likely to be the best-sellers in shops serving some of the UK's most deprived communities. Thus MUP is likely to impact on the household budgets of poorest drinkers the most. These are the groups who currently experience the greatest levels of alcohol-related (and other) health inequalities. However, it remains to be seen how these inequalities will change post-MUP. It may reduce alcohol problems amongst the poorest drinkers, but it might also intensify the underlying economic hardships predictive of ill-health (as with higher cigarette excise taxes, Farrelly *et al*., 2012). It may reduce consumption by problem drinkers, but in doing so risk exacerbating the hardships felt by their families (recent research suggests childhood economic hardship predicts adulthood alcohol problems, but not adulthood consumption, Gauffin *et al*., 2013).

MUP is certain to influence alcohol product choice or brand preference. Post-MUP, products such as white cider should become less competitive, while others such as the caffeinated ‘tonic’ wine are likely to become more tempting (with consequently less plastic and more glassware in the hands of ‘street drinkers’, Forsyth *et al*., 2010). The consequences of MUP for the retail sector also need to be assessed. For example, will the shops who participated in this research benefit from MUP, and, especially if they do not, how this might influence patterns of community shopping provision and hence local health-related environments. Assuming MUP is enacted in Scotland, the present study provides a baseline for future research to address these questions.

## Conflict of interest statement

None declared.
